# Tsetse GmmSRPN10 Has Anti-complement Activity and Is Important for Successful Establishment of Trypanosome Infections in the Fly Midgut

**DOI:** 10.1371/journal.pntd.0003448

**Published:** 2015-01-08

**Authors:** Cher-Pheng Ooi, Lee R. Haines, Daniel M. Southern, Michael J. Lehane, Alvaro Acosta-Serrano

**Affiliations:** 1 Department of Life Sciences, Sir Alexander Fleming Building, Imperial College-South Kensington, London, United Kingdom; 2 Department of Vector Biology, Liverpool School of Tropical Medicine, Liverpool, United Kingdom, 3 Department of Parasitology, Liverpool School of Tropical Medicine, Liverpool, United Kingdom; National Institute of Allergy and Infectious Diseases, United States of America

## Abstract

The complement cascade in mammalian blood can damage the alimentary tract of haematophagous arthropods. As such, these animals have evolved their own repertoire of complement-inactivating factors, which are inadvertently exploited by blood-borne pathogens to escape complement lysis. Unlike the bloodstream stages, the procyclic (insect) stage of *Trypanosoma brucei* is highly susceptible to complement killing, which is puzzling considering that a tsetse takes a bloodmeal every 2–4 days. In this study, we identified four tsetse (*Glossina morsitans morsitans*) serine protease inhibitors (serpins) from a midgut expressed sequence tag (EST) library (*GmmSRPN3*, *GmmSRPN5*, *GmmSRPN9* and *GmmSRPN10*) and investigated their role in modulating the establishment of a *T. brucei* infection in the midgut. Although not having evolved in a common blood-feeding ancestor, all four serpins have an active site sharing remarkable homology with the human complement C1-inhibitor serpin, SerpinG1. RNAi knockdown of individual *GmmSRPN9* and *GmmSRPN10* genes resulted in a significant decreased rate of infection by procyclic form *T. brucei*. Furthermore, recombinant *GmmSRPN10* was both able to inhibit the activity of human complement-cascade serine proteases, C1s and Factor D, and to protect the *in vitro* killing of procyclic trypanosomes when incubated with complement-activated human serum. Thus, the secretion of serpins, which may be part of a bloodmeal complement inactivation system in tsetse, is used by procyclic trypanosomes to evade an influx of fresh trypanolytic complement with each bloodmeal. This highlights another facet of the complicated relationship between *T. brucei* and its tsetse vector, where the parasite takes advantage of tsetse physiology to further its chances of propagation and transmission.

## Author Summary

Blood feeding arthropods are exploited by blood borne parasites as vectors of transmission. *Trypanosoma brucei*, a salivarian trypanosome species, must survive, migrate and differentiate in the tsetse until they become mature, mammalian-infective forms within the fly salivary glands. This constitutes a significant challenge to trypanosomes as the major parasite form colonising the tsetse midgut is sensitive to lysis by blood complement, which is introduced into the tsetse gut whenever the fly feeds. In this study, we show that *T. brucei* may avoid being eliminated by bloodmeal complement by benefitting from a complement-inhibiting enzyme secreted by the fly itself. We showed that this serine protease inhibitor (serpin) enzyme, Serpin10, can inactivate triggers of the complement cascade, protect tsetse-infective trypanosomes from complement lysis, and is important for trypanosome establishment in the tsetse midgut. Taken together, we propose that GmmSRPN10 may be part of a repertoire of complement-inhibiting proteins secreted by tsetse that are utilized by *T. brucei* to evade complement lysis in the tsetse midgut.

## Introduction

Mammalian blood constitutes a challenging source of nutrition, yet a multitude of arthropods have evolved to exploit this survival niche [1]. Besides developing physical and behavioural adaptations to evade their hosts, haematophagous arthropods have also adapted physiologically to ingest a food source that is relatively poor in vitamins and spiked with a cocktail of immunity factors. This cocktail also contains factors involved in the complement cascade, which is activated and mediated by a series of serine proteases [2]. Activated by either the classical, alternative or lectin pathways, the cascade leads to the formation of pores across the plasma membrane of targeted cells. These pores ultimately cause the disruption of cellular compartmentalisation and lead to cell lysis.

Complement-induced cytolysis is detrimental to both blood-borne pathogens and blood-feeding arthropods alike. Unicellular parasites are rapidly lysed upon exposure [3], [4], while blood-feeding arthropods can acquire gut damage as a consequence of ingesting a bloodmeal containing active complement [5], [6]. As the complement cascade is activated and mediated by a series of serine proteases [7], haematophagous arthropods have evolved molecular counter-measures against complement-mediated damage by secreting serine protease inhibitors (serpins) to suppress cascade activation [8]–[10]. Serpins can have multiple functions within arthropods, ranging from immunity to development [11]–[14], including inactivation of serum complement as a means to prevent damage to the alimentary canal of haematophagous arthropods [8], [15]. As haematophagy is frequently associated with pathogen transmission, it is unsurprising that transmitted pathogens can hijack the anti-complement defences of their arthropod vector to better improve their chances of survival [9], [16].

*Trypanosoma brucei*, a causative agent of African trypanosomiasis, has an alternating life cycle between mammalian hosts and the tsetse fly. *T. brucei* can only be transmitted to other vertebrate hosts by the infective bite of a tsetse. To accomplish transmission, the parasite must first establish an infection in the fly midgut before it can attempt a long and complex migratory process (including crossing the midgut ectoperitrophic matrix on two occasions) to finally establish a mature infection as metacyclic trypomastigotes in the salivary glands (SG) [17]–[19]. Bloodstream form *T. brucei* is partially immune to serum complement due to the rapid clearance rate of bound molecules from its cell surface [20], [21], however it becomes susceptible to complement lysis upon differentiating to the procyclic insect form [22]. In the tsetse midgut, an influx of fresh, complement-rich blood occurs when the fly feeds every 2–4 days and involves bloodmeals weights far exceeding that of the fly [23]. Besides exposing the tsetse midgut to active complement, serum complement is also detrimental to any trypanosome procyclic forms (PFs) already residing within the tsetse midgut. It is currently unresolved how these parasites survive this repeated exposure to trypanolytic blood.

Here we examined the role of four *Glossina morsitans morsitans* serpins (*GmmSRPN*s), first identified from an EST screen to localise to the tsetse midgut [24]. We found these tsetse serpins to share significant sequence identity of the active site with the innate serpin of the mammalian complement cascade (Serpin G1), and postulated that they might be expressed by the tsetse to inhibit complement cascade components in mammalian serum. Knockdown of these serpins resulted in reduced trypanosome infection rates in tsetse flies and we show that one of them, GmmSRPN10 can directly inhibit the activity of complement cascade serine proteases. Taken together, our findings suggest that tsetse serpins can act to rapidly inhibit bloodmeal complement, which inadvertently protects PF trypanosomes as they establish an infection in the midgut.

## Methods

### Generation of phylogenetic tree

A list of insect serpins from GenBank (NCBI) was compiled from insect species with sequenced genomes [25]: *Aedes aegypti*, *Anopheles gambiae*, *Apis mellifera*, *Bombyx mori*, *Drosophila melanogaster* and *Tribolium castaneum*. COBALT (NCBI) was used to generate an initial alignment of extracted serpin sequences. Clusters of related serpins were then further aligned and analysed using CLC sequence viewer using the neighbour-joining algorithm with the bootstrap replicate value set for 100.

### Cultured trypanosomes

Bloodstream form (BSF) *Trypanosoma brucei brucei* strain MSUS/CI/78/TSW 196 [26] was transformed into procyclic culture forms (PFs) by suspension in DTM medium with a final *cis*-aconitate (Sigma) concentration of 3 mM and cultured at 26°C [27]. Transformed trypanosomes were initially subcultured and maintained in a 1∶1 mix of DTM and SDM-79 media (Gibco), but subsequently cultured with SDM-79.

### Infection of tsetse with *T. brucei*

For infection experiments utilising horse serum (HS), HS was extracted from each batch of defibrinated blood by centrifugation and frozen in aliquots to prevent loss of complement activity from repeated freeze thawing. Unfed (teneral) flies were infected with TSW 196 BSFs suspended in serum (untreated or treated either with HI or CVF) 48 h post emergence and maintained on the same serum feed until dissection 10 days post-infection.

### Generation of dsRNA

PCR amplification of the genes for knockdown experiments (*GmmSRPN3, GmmSRPN5, GmmSRPN9, GmmSRPN10*) used pE*GFP-N1 (CLONTECH Laboratories, USA)* as the nonspecific dsRNA control, were carried with the following primer pairs with a preceding *T7* RNA polymerase promoter sequence (underlined) for use with the MEGAscript RNA transcription kit (Ambion) using plasmid template from a tsetse midgut EST library <annotated> [24]:

***GmmSRPN3*** <Gmm-2356>

Forward: TAATACGACTCACTATAGGGCTGGGGGAGGGCGACAAGA

Reverse: TAATACGACTCACTATAGGGTTCCCTGGGCAAAATAATGAGCA

***GmmSRPN5*** <Gmm-3352>

Forward: TAATACGACTCACTATAGGGAACAGCAGTACGAGCGGATTTAT

Reverse: TAATACGACTCACTATAGGGTTGGCCCATTGACCTTTGA

***GmmSRPN9*** <Gmm-72e08>

Forward: TAATACGACTCACTATAGGGCTACGCGAGGCTTACTTCC

Reverse: TAATACGACTCACTATAGGGCGCCTCGGTCCCTTCTTCAT

***GmmSRPN10*** <Gmm-3334>

Forward: TAATACGACTCACTATAGGGTCCGCTGATTGTTTTGACTCG

Reverse: TAATACGACTCACTATAGGGCCGCTTCCGTACCTTCCTC

***Cloning vector pEGFP-N1***

Forward: TAATACGACTCACTATAGGGACGTAAACGGCCACAAGTTC

Reverse: TAATACGACTCACTATAGGGCTTGTACAGCTCGTCCATGCC

### Scoring of infection rates in dsRNA knockdown flies

The *Glossina morsitans morsitans* colony (LSTM) was reared at 24–27°C, 68–78% humidity and maintained by membrane feeding on defibrinated horse blood (TCS Biosciences). Teneral flies were fed dsRNA-spiked blood at a concentration of 330 µg/ml (∼10 µg dsRNA ingested per fly as previously reported [28]. Flies fed on dsRNA were infected in the second bloodmeal (48 h post dsRNA feed) with *T. brucei* TSW 196 BSFs and dissected 10 days post infection. The midguts were analysed by microscopy for the presence of trypanosomes and tissue samples were flash frozen in liquid nitrogen and stored at −80°C until required for RT-PCR or qPCR analyses and Western blotting analysis.

### Determination of global serpin transcript levels by semi-quantitative RT-PCR

Total RNA from flash frozen samples was extracted using Trizol (Invitrogen) and standardised to a concentration of 25 ng/µl. cDNA was generated using the AccessQuick RT-PCR kit (Promega) according to the manufacturer's recommendations and subsequent PCR was carried out with primers used to generate dsRNA, as well as primers for tsetse glyceraldehyde-3-phosphate dehydrogenase (*GAPDH*):

### GAPDH

Forward: TAAAATGGGTGGATGGTGAGAGTC

Reverse: CTACGATGAAATTAAGGCAAAAGT

RT-PCR products were visualised on a 1% (w/v) agarose gel and transcript levels were expressed as percentage against the band intensity (SynGene) for *GAPDH* controls.

### Recombinant expression of His::GmmSRPN10

The coding region for *GmmSRPN10* was amplified from the *G. m. morsitans* midgut EST library clone [25] using PCR (New England Biolabs, UK) according to the manufacturer's recommendations with primers having *PmlI* and *SacI* restriction enzyme overhangs (underlined).

Forward primer: TTTCACGTGATGTCGGATTTAAATTTACAA;

Reverse primer: TTTGAGCTCTTAAGCGTCTGGTGCGTTAAC.

Poly-A tailing was performed using reagents from the NEB PCR kit and ligated into a pGEM-T Easy holding vector (Promega). Holding vectors were transformed into *E. coli* XL-1 cells. Plasmid extraction from cultured XL-1 cells plus holding vector was performed using a miniprep kit (Qiagen). Extracted plasmids were subjected to *Pmll* and *Sacl* restriction enzyme (Promega) digest and the digestion products run on a 1% (w/v) agarose gel. The agarose gel bands corresponding to *GmmSRPN10* coding region with cut restriction sites were excised from the gel and purified using the PureLink Gel Extraction Kit (Invitrogen). Gel purified bands were ligated into the pET-45b expression vector (Novagen), which allows for a His-tagged expression product. The expression construct was transformed into an *E. coli* Rosetta-gami (DE3)pLysS expression cell line (donated by Mark Paine, LSTM).

### Expression and purification of His::GmmSRPN10

Transformed Rosetta-gami cells were grown in ampicillin LB (50 µg/L) at 37°C to an OD_595_ of 0.5–0.8. Expression was induced by isopropyl-1-thio-β-D-galactopyranoside at a final concentration of 1 mM. Expression continued for 24 h at 30°C with agitation. Cells were harvested by centrifugation at 3,500× *g* for 15 min at 4°C and reconstituted in PBS. Harvested cells were sonicated on ice at 30 s intervals (with intermittent cooling periods of 30 s). Cellular debris were removed by centrifugation at 3,500 *g* for 15 min at 4°C and the supernatant was purified with a Ni-NTA agarose column (Qiagen) by selectively binding the His-tagged recombinant protein at an imidazole concentration of 20 mM and a NaCl concentration of 0.25 mM. Bound protein was first washed with wash buffer (50 mM sodium phosphate, 300 mM NaCl, 20 mM imidazole) and then eluted off the column with elution buffer (50 mM sodium phosphate, pH 8.0, 250 mM imidazole). The identity of the recombinant protein was confirmed using SDS-PAGE and mass spectrometry after in-gel trypsinisation and LC-MS/MS analysis (at the University of Dundee Post-Genomics Facility).

Confirmation of inhibitory activity of purified His::Serpin10 was carried out by assaying its inhibition of bovine pancreatic trypsin (Sigma) activity in the presence of Nα-benzoyl-L-arginine ethyl ester (BAEE) substrate according to manufacturer's protocol (Sigma) with slight modifications. Briefly, 100 BAEE units of trypsin was incubated with His::Serpin10 for 10 min at room temperature prior to addition of BAEE. The absorbance (253 nm) of the reaction mix was then continuously recorded for 5 min or until saturation of the absorbance value. Trypsin activity was subsequently calculated from the maximum linear rate of the increase in absorbance (253 nm).

### Trypanosome lysis assays

Complement assays were performed at 26°C for 30 min in 200 µL volumes with 25% (v/v) concentration of horse serum (HS) containing 10^6^/ml PF trypanosomes. Assays were stopped with 300 µL of cold SDM-79 medium. Surviving trypanosomes were counted on a Neubauer haemocytometer. The % survival was normalised against PFs incubated with heat-inactivated (HI) serum (56°C for 1 h), with the exception of assays where serum was inactivated using His::GmmSRPN10. In these experiments, the % survival was normalised against PFs exposed to serum pre-treated with heat denatured (100°C for 10 min) His::GmmSRPN10. The complement assay was also used to determine the degree of complement inactivation caused by cobra venom factor (Quidell) or bovine trypsin (Sigma).

### rC1s and rFactorD inhibition assays

rC1s and rFactorD (R&D Systems) were assayed according to manufacturer's instructions with slight modifications. Briefly, His::GmmSRPN10 was assayed for its ability to inhibit 5 ng/µl of rFactorD or rC1s activity using a 5,5′Dithio-bis-2-nitrobenzoic acid (Sigma) Z-Lys-SBzl (Bachem) reporter system with increasing concentrations of His::GmmSRPN10. Reaction mixes were prepared on a 96 well plate with absorbance (405 nm) measured over 20 minutes in a plate reader.

### Generation of GmmSRPN10 antisera for western blotting

*In silico* prediction of target peptide antigenicity, solubility and specificity was done using SVMTriP [29] and a high scoring epitope (QTIKDDFWISSEESVQLEYM) was chosen for its specificity to GmmSRPN10 (GMOY012007) via reciprocal BLAST with Vectorbase [30]. The key antigenic region of the epitope (underlined) was predicted using an online resource by the Universidad Complutense de Madrid utilising the Kolaskar and Tongaonkar method [31]. The final peptide sequence (KDDFWISSEESVQLEY) was used to immunise rabbits (EurogenTec) to generate antisera specific for GmmSRPN10 (α-GmmSRPN10). The polyclonal rabbit antisera was subsequently affinity purified for IgG using a Nab Spin Kit (Fisher Scientific UK Ltd) to remove cross-reactivity with blood fed midgut samples. Western blots using anti-GmmSRPN10 at 1∶400 dilution and goat α-rabbit HRP-conjugated secondary antibody (Thermo Scientific) at 1∶25,000 dilution were developed using SuperSignal West Dura (Pierce) substrate and Carestream® Kodak BioMax Light film (Sigma). All blot membranes were subsequently stained with 0.2% (w/v) nigrosine in PBS (Sigma) to confirm equal protein loading.

### qPCR analyses of *GmmSRPN10* mRNA from tsetse midgut tissue

cDNA was prepared from Trizol-extracted total RNA using AccuScript Reverse Transcriptase (Agilent Technologies) or with SuperScript III Reverse Transcriptase (Life Technologies). qPCR was performed using Brilliant III Ultra-Fast SYBR Green QPCR Master Mix (Agilent Technologies) and primers specific for *GmmSRPN10*, tsetse *β-tubulin* and tsetse *α-tubulin*, using a Strategene Mx3005P real time PCR detection system (Agilent Technologies). Analysis for fold change in dsGmmSRPN10-fed tsetse compared to dsEGFP-fed tsetse was carried out using the Pfaffl method [32] with *α-tubulin* as the reference gene for *GmmSpn10* and *β-tubulin*. Primer sequences for *β-tubulin* were taken from [33], while the primers for *α-tubulin and GmmSRPN10* are:

GmmSRPN10 Forward – TACTTCCAATTCGGCACCAG

GmmSRPN10 Reverse – CCTGAATACGCAAACCTCGT

### Statistical analyses

Non-parametric t-test and one way ANOVA were carried out with GraphPad Prism5, with *P<0.05* considered significant and *P<0.001* considered highly significant. All error bars presented in figures represent standard deviation.

## Results

### Four putative serpins were identified from the tsetse midgut EST library

**Figure 1:**
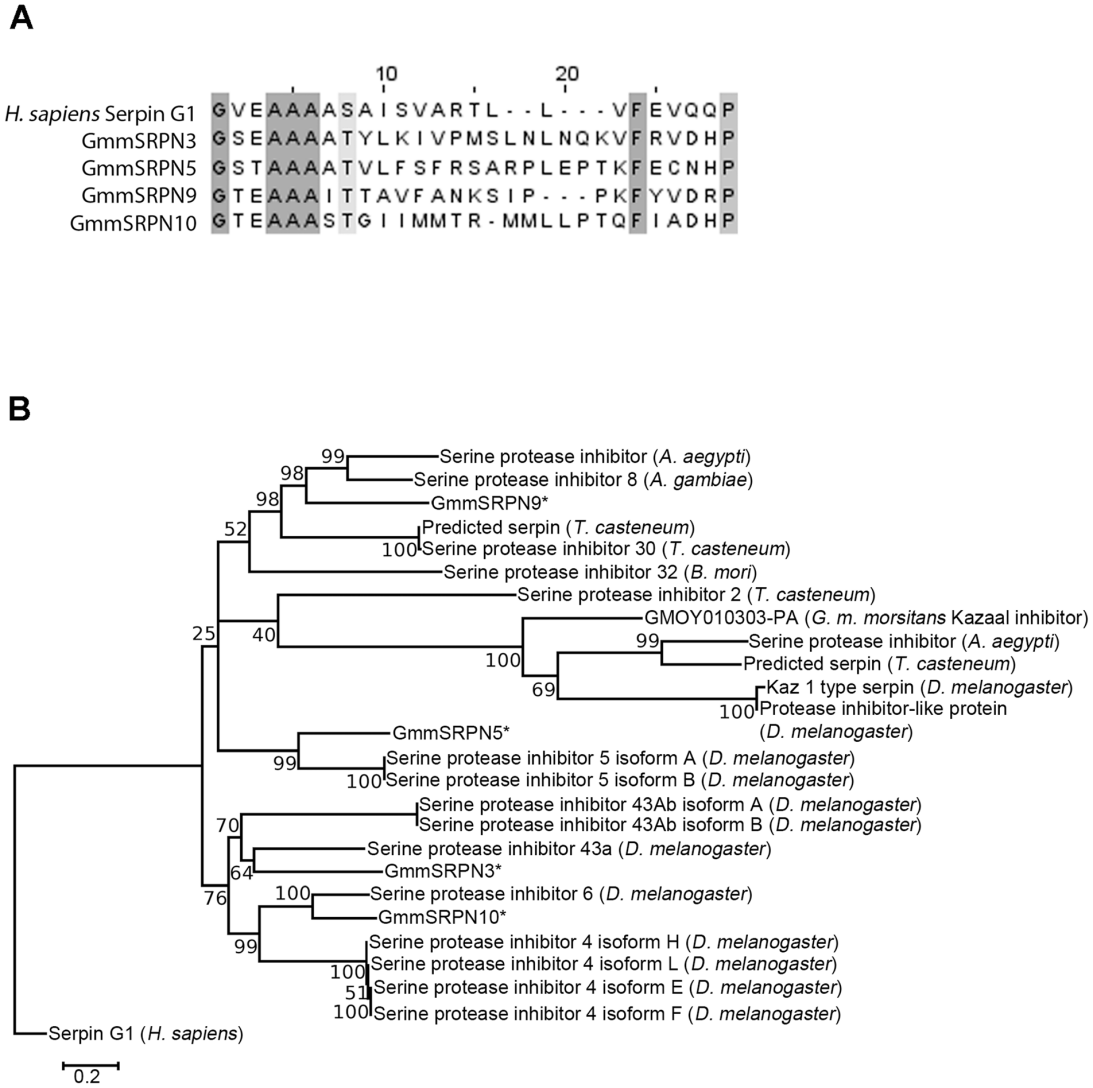
Putative tsetse midgut serpins show active site conservation with human complement cascade inhibitory serpin, but may not have evolved as a consequence of a haematophagous lifestyle. (A) Clustal-W alignment the putative tsetse serpins RCL region (GmmSRPN3, GmmSRPN5, GmmSRPN9, GmmSRPN10) with human SerpinG1 complement cascade inhibitor indicate conserved (highlighted in grey) key residues involved in the inactivation of serine proteases. (B) A phylogenetic tree (with bootstrap values shown) generated from neighbour-joining alignment of putative tsetse midgut serpins (denoted with *) and representative serpins from arthropod species with fully sequenced genomes (*Aedes aegypti*, *Anopheles gambiae*, *Apis mellifera*, *Bombyx mori*, *Drosophila melanogaster* and *Tribolium castaneum*). Distinct clustering of these putative midgut serpins with that of haematophagous species (*Aedes aegypti, Anopheles gambiae*) was not apparent, suggesting that the serpins may have either evolved following speciation of *Glossina morsitans morsitans* or from the last common arthropod ancestor.

As an annotated genome was not available at the commencement of this research, the *G. m. morsitans* EST library was used to identify four putative serpins (Table 1) from midgut tissue [24]. An alignment of the active site of these serpins with that of the human complement cascade inhibitor SerpinG1 using Clustal-W (Fig. 1A) showed key residues were conserved [34] within the reactive centre loop (RCL). As the RCL constitutes the active site of serpins, we postulated that these four serpins may have complement cascade inhibiting properties associated with a haematophagous diet.

**Table 1:** Investigated tsetse midgut serpins.

VectorBase genome designation		PubMed EST designation		Number of amino acids
VectorBase GMOY annotation	GMOY ID	PubMed annotation	GeneBank accession number	
Serpin 3	GMOY002444	Serpin 1	ABC25072	406
Serpin 5	GMOY000990	Serpin 5	ABC25075	421
Serpin 9	GMOY000930	Serpin	ABC25079	437
Serpin 10	GMOY012007	Serpin 6Serp2	ABC25076AFG28184	240376

Four putative serpins were initially identified from a tsetse midgut EST library. Subsequent sequencing and annotation of the genome has now assigned these serpins with GMOY IDs in the Invertebrate Vectors of Human Pathogens database, VectorBase (https://www.vectorbase.org).

A phylogenetic tree (Fig. 1B) was generated from neighbour-joining alignments [35] for serpins selected from fully-sequenced arthropod genomes. To determine if these serpins were associated with blood-feeding, we included representatives of blood-feeding arthropods (*Aedes aegypti* and *Anopheles gambiae*) and non-blood feeders (*Drosophila melanogaster and Tribolium castaneum*). This did not result in a clear distinction between serpins evolved as a consequence of haematophagy. To determine if adaptation to blood feeding directly applies evolutionary pressure to the RCL of insect serpins, phylogenetic trees were also generated from a subset of these representative serpins using either full length protein sequence (S1A Fig.) or RCL sequence (S1B Fig.). Likewise there was no clear clustering of serpins associated with adaptation to haematophagy in either analysis. This is perhaps due to the high degree of conservation displayed across serpin proteins [36], [37].

### Knockdown of tsetse serpins decreases trypanosome infection rate

We reasoned that should these tsetse serpins have complement inhibiting properties, knocking them down in tsetse may be detrimental to PF trypanosomes establishing in the midgut. Gene knockdown in tsetse can be achieved by either injection or feeding of dsRNA [28]. To prevent activation of fly immunity by puncturing its cuticle, the feeding route was chosen. Feeding dsRNA led to consistent knockdown of the target gene compared to control flies fed with dsRNA targeting *EGFP* (S2 Fig.). Monitoring of gene knockdown was carried out on the transcript level by semi-quantitative RT-PCR, which allowed for a rapid overview of gene knockdown and its associated infection phenotype.

**Figure 2:**
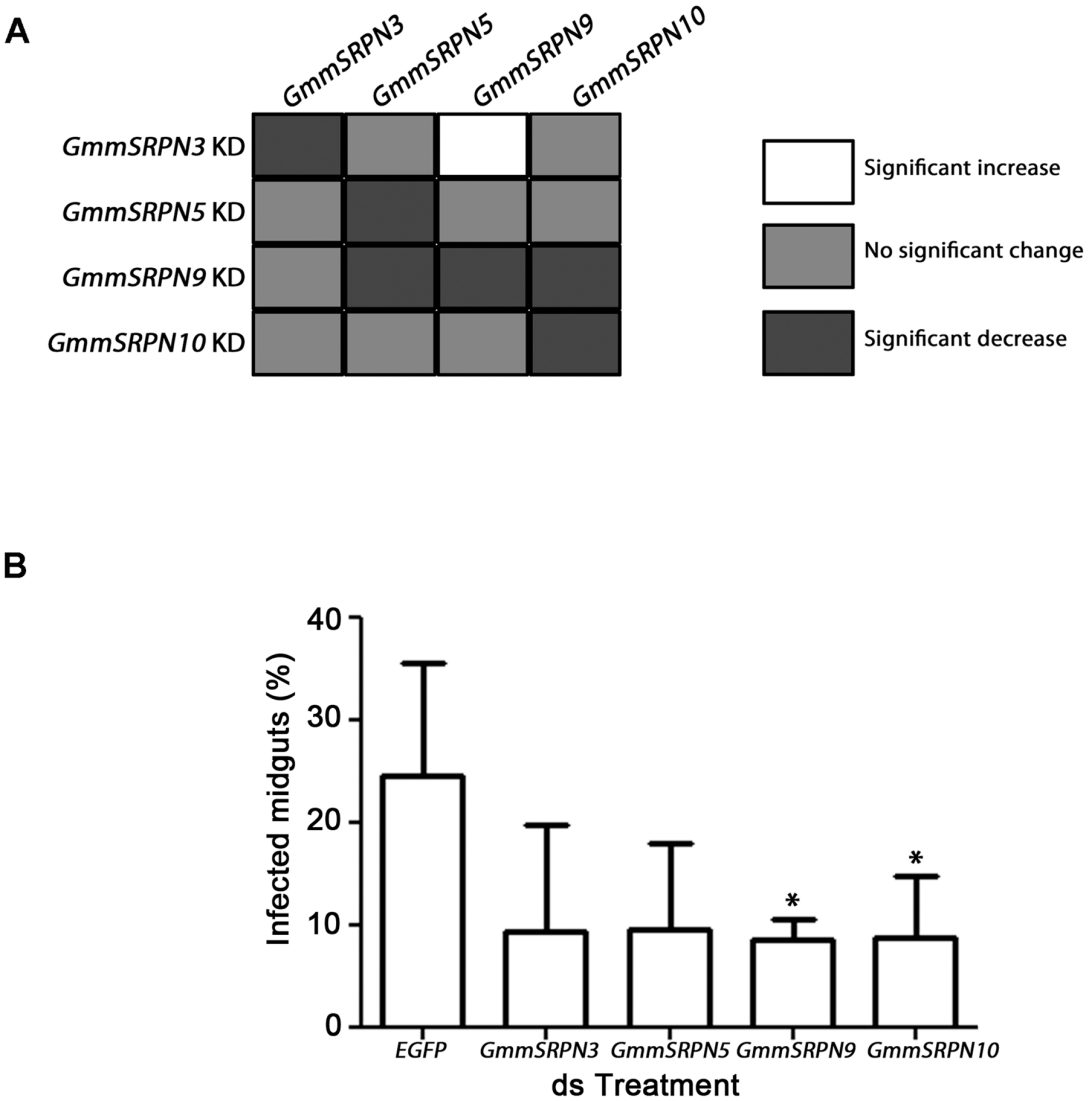
dsRNA knockdown of putative tsetse serpins results in decreased trypanosome prevalence in the tsetse midgut. (A) Individual knockdown (KD) of putative tsetse midgut serpins by feeding dsRNA resulted in the suppression of the target *serpin* mRNA transcript. A degree of cross-reactivity was observed with two genes, with knockdown of *GmmSRPN9* leading to a significant decrease in transcript level of two other serpins (*GmmSRPN5* and *GmmSRPN10*) and knockdown of *GmmSRPN3* leading to a significant increase in *GmmSRPN9*. (B) Knocking down each putative tsetse midgut serpin resulted in a decrease in trypanosome infection rates in the tsetse midgut ten days post infection, but this decrease in infection rate was only significant when either *GmmSRPN9* or *GmmSRPN10* were knocked down compared to ds*EGFP*-treated controls (P<0.05, t-test and one-way ANOVA). Knockdowns were carried out in tandem with approximately 20 flies per group and each bar represents at 3 biological replicates.

There was variability in band intensity from our RT-PCR experiments and we have therefore highlighted changes of statistical significance from 3 biological replicates (S1 Table) in Fig. 2A. Using this criteria, a degree of cross reactivity was observed with certain gene targets. There was co-suppression in the case of *GmmSRPN9 knockdown*, but also up-regulation when *GmmSRPN3* was targeted. The co-knockdowns observed with *dsGmmSRPN9* was unlikely to be due to non-specific targeting of the generated dsRNA as there was no continuously homologous regions of the expected dsRNA sequences with non-target serpins (S2 Table). This, coupled with the up-regulation observed with *GmmSRPN9* when *GmmSRPN3* was targeted suggests that this phenomenon may be due to a physiological response by the tsetse.

Regardless of which serpin was targeted, gene knockdown led to a decrease in trypanosome infection rate within the tsetse midgut (Fig. 2B). This was on average half the infection rate expected of a second bloodmeal infection [38]. In this preliminary experiment, the decrease in infection rate was significant when either *GmmSRPN10* or *GmmSRPN9* were targeted. As illustrated by the co-repression and up-regulation events going on with certain serpin knockdowns, we concluded that it would be difficult to determine gene function via knockdown alone. Furthermore, serpins involved in insect innate immunity may also confer infection phenotypes upon trypanosome elimination [39]. Therefore, we next attempted to express recombinant serpins to further elucidate their function with *in vitro* biochemical assays.

### Active complement in horse serum is lethal to *T. brucei* PFs

**Figure 3:**
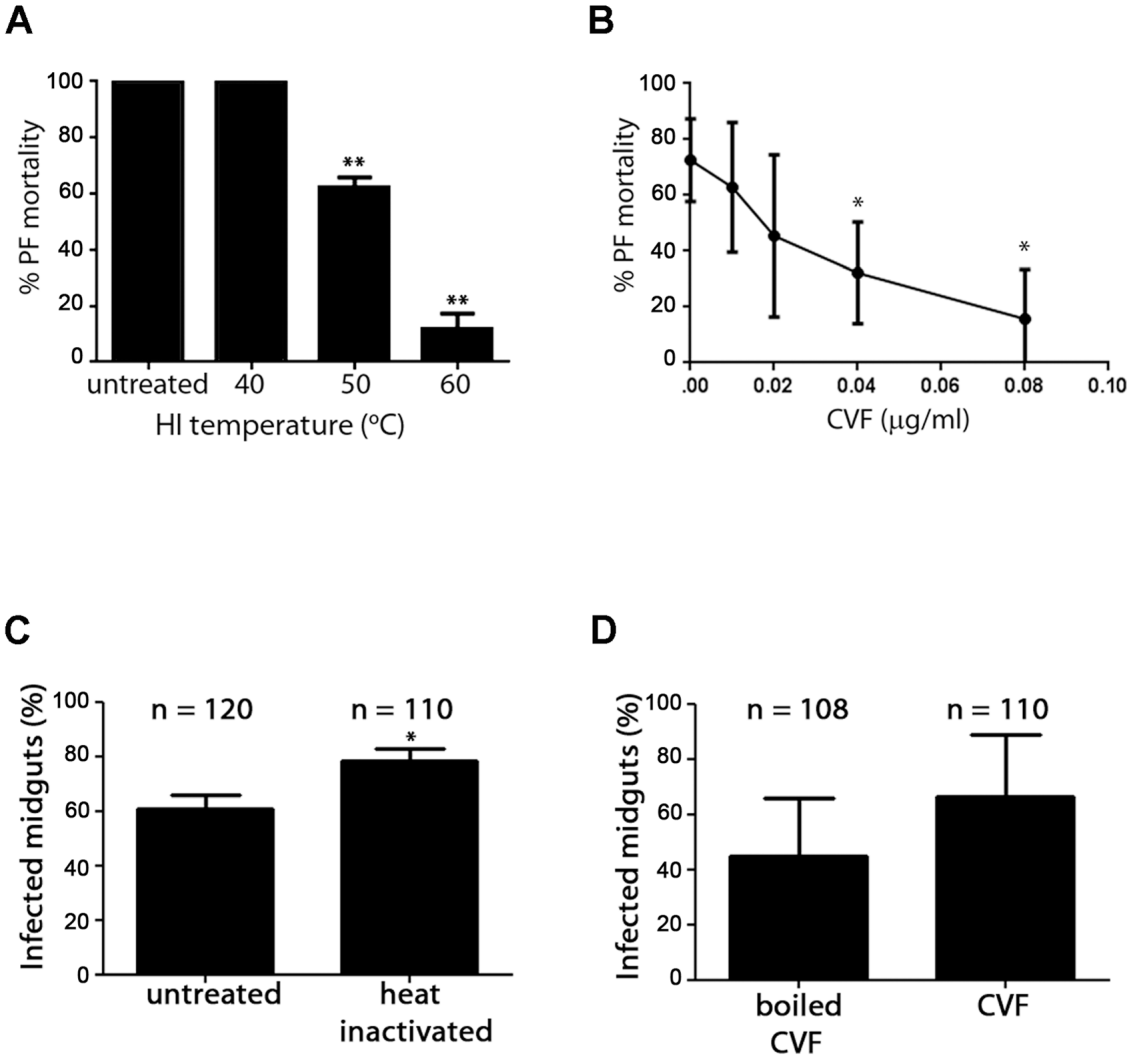
HS complement is lethal to PFs. (A) The lethality of HS can be inactivated by pre-treating the serum with heat. PF mortality decreases significantly when HS is heat inactivated at 50°C for 1 h, and up to 88.5±5.67% survive when the temperature of inactivation was 60°C. (B) PF lysis by HS decreases when HS was pre-treated with CVF to exhaust complement cascade factors. (C) Tsetse infection experiments carried out using heat inactivated serum resulted in a significant (P = 0.01) increase in infected midguts in experimental groups where heat inactivated serum was used for infection and feeding. (D) A similar increase in infection rate was observed when the HS used in experiments was pre-treated with CVF, though this increase was not statistically significant compared to tsetse that were infected and maintained with HS pre-treated with boiled CVF after 4 experimental replicates.

HS is trypanocidal to trypanosome PFs. This activity can be inhibited by pre-treating the serum with heat inactivation (HI) or cobra venom factor (CVF). CVF exhausts the complement cascade by an overdrive mechanism where the cascade components are consumed and activated in the absence of an activating antigen [40], [41]. Complement-inactivated HS prevents lysis of trypanosomes *in vitro* (Fig. 3A & B), demonstrating that the complement cascade is the lethal component in HS. When complement-inactivated HS was used for tsetse infection experiments, groups where *T. brucei* infection and subsequent feeding were carried out using complement-inactivated HS had an increase in *T. brucei* infection rate (Fig. 3C & D). This increase was statistically significant (P = 0.01) when the HS was inactivated by HI but not by CVF. Taken together, these data suggests that the complement component of HS is lethal towards *T. brucei* PFs, a phenomenon that remains relevant in the context of infection.

### Recombinant GmmSRPN10 has complement-inhibiting activity

To facilitate a better understanding of the function of midgut serpins in *G. m. morsitans*, we attempted to express recombinant proteins using a bacterial expression system with the pET-45b expression vector. Addition of a histidine tag (His-tag) on the N-terminus of the recombinant proteins permitted purification by a nickel-affinity column without interfering with the RCL on the C terminus. All attempts to express recombinant proteins using BL21(DE3) failed, perhaps due to the extent of rare eukaryote-specific codons present (S3 Table). However, recombinant expression was possible for *GmmSRPN10* using a Rosetta-gami *E. coli* cell line as this bacterial strain allows for expression of proteins with rare eukaryotic codons.

**Figure 4:**
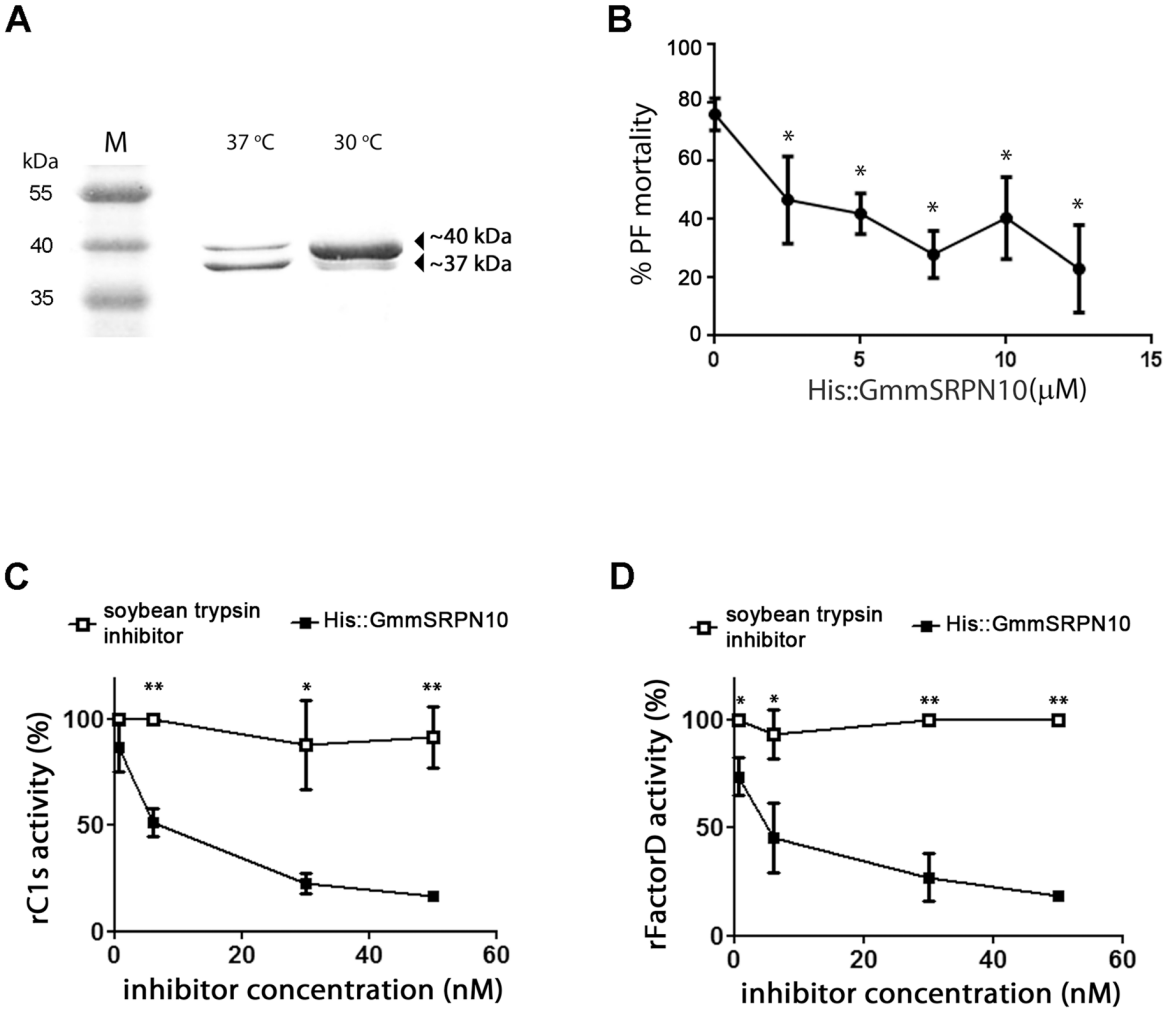
Expression of His::GmmSRPN10 is temperature sensitive and the expressed protein inhibits killing of PF trypanosomes by HS *in vitro*. (A) Recombinant expression of His::GmmSRPN10 results in an intact (∼40 kDa) and truncated (∼37 kDa) protein fraction that were identified using mass spectrometry. Expression at 30°C enriches the intact fraction. (B) Supression of trypanocidal activity by His::GmmSRPN10 can be achieved by pre-treating HS with the recombinant protein. (C–D) His::GmmSRPN10 inhibits the activity of rC1s and rFactorD, recombinant human complement cascade serine proteases, in a concentration dependent manner. Inhibition of the complement cascade serine proteases at comparable concentrations were not observed in the controls performed in tandem using a commercially available soybean trypsin inhibitor.

Elution of His::GmmSRPN10 from a nickel column produced two proteins of similar apparent molecular mass (∼40 kDa and ∼37 kDa) as determined by Coomassie staining of SDS-PAGE gels (Fig. 4A). The relative abundance of the two eluted proteins was dependent on the temperature at which expression was carried out, with a higher proportion of the higher molecular weight band produced when the expression temperature was set at 30°C compared to when expression was carried out at 37°C. Identification of excised bands by trypsin digest followed with mass spectrometry revealed the ∼40 kDa band to be His::GmmSRPN10 while the ∼37 kDa product was truncated His::GmmSRPN10 lacking the C-terminus RCL (S3A Fig.). This indicates that the stability of His::GmmSRPN10 is either temperature sensitive or susceptible to bacterial heat shock proteins.

His::GmmSRPN10 inhibits the trypanocidal activity of HS in a concentration dependent manner (Fig. 4B), suggesting that His::GmmSRPN10 inactivates the complement cascade in HS. Increasing concentrations of His::GmmSRPN10 were able to inhibit the activity of bovine trypsin (S3B Fig.), thus we reasoned that the RCL of His::GmmSRPN10 was functional and may be targeting complement cascade-specific serine proteases.

We next tested the inhibitory activity of His::GmmSRPN10 against two commercially available human complement cascade serine proteases, recombinant (r) C1s and Factor D. C1s activates the classical complement pathway and Factor D activates the alternative pathway. rFactor D was chosen for this analysis as complement lysis of *T. brucei in vitro* is activated via the alternative pathway [22]. rC1s was also included as we reasoned that a serpin that inhibits complement cascade serine proteases may not be pathway specific. His::GmmSRPN10 blocked the activity of both rC1s and rFactorD in a concentration dependent manner (Fig. 4C & D) compared to a commercial trypsin inhibitor at comparable concentrations. Taken together, these data suggests that GmmSRPN10 is an inhibitory serpin that inactivates complement killing of PF trypanosomes within the tsetse midgut.

### GmmSRPN10 is a secreted protein important for PF survival in the tsetse midgut

**Figure 5:**
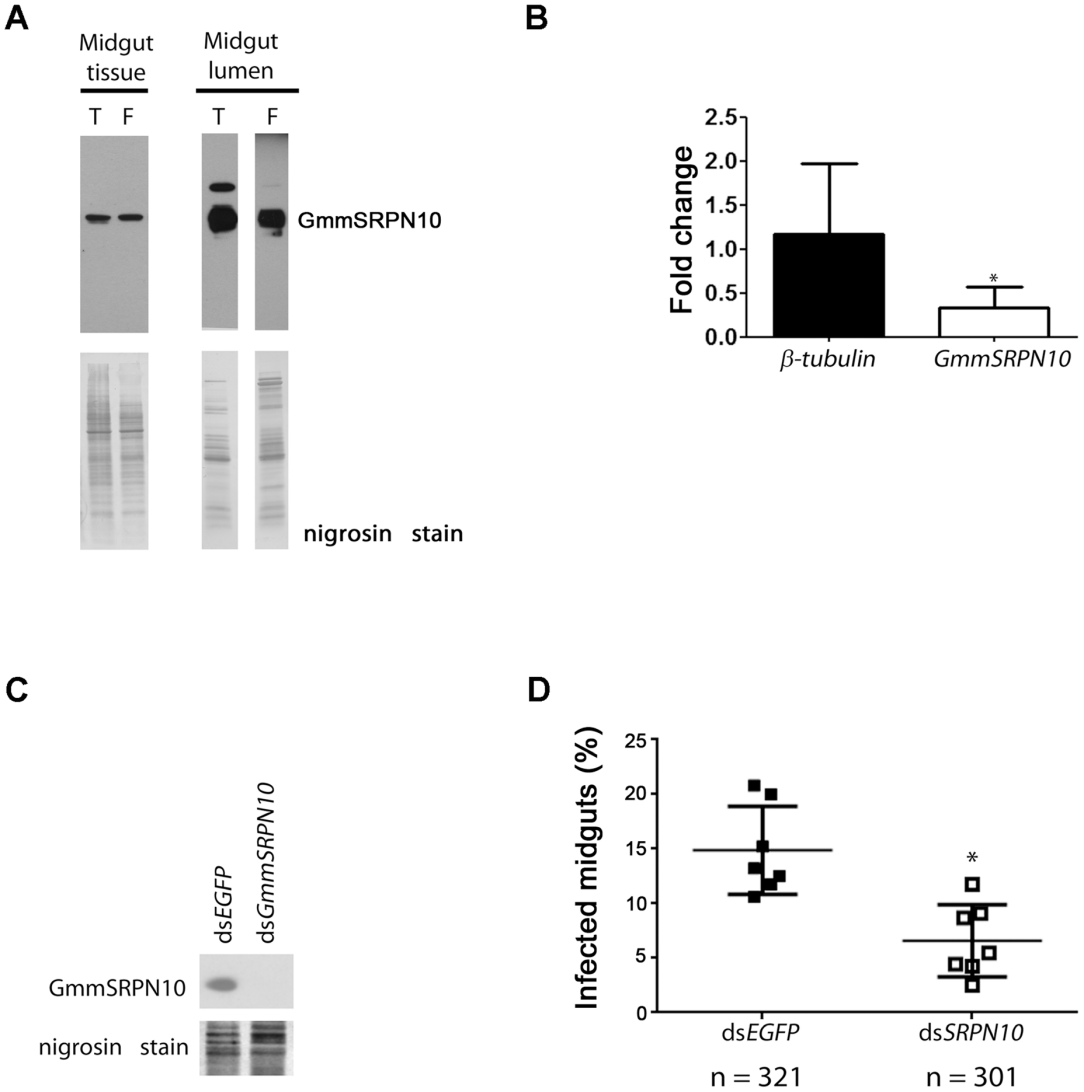
GmmSRPN10 is secreted into the midgut lumen and is important for PF infection. (A) Western blot (using α-GmmSRPN10 antisera) analyses of midgut tissue and midgut lumen content from teneral (T) and fed (F) tsetse suggests that GmmSRPN10 (arrow) is secreted into the midgut lumen in teneral (newly emerged and yet unfed) flies in preparation for blood feeding. Lower panel, stained PVDF membrane with nigrosine. (B) Comparison of *β-tubulin* and *GmmSRPN10* transcript levels between *GmmSRPN10* knockdown and control tsetse at the point of dissection at ten days post trypanosome infection indicate that *β-tubulin* transcript levels remain relatively unchanged in knockdown flies while *GmmSRPN10* transcript is significantly decreased to less than 50% (bars represent 3 independent experiments). (C) Downregulation of GmmSRPN10 protein in midguts from dsGmmSRPN10 treated knockdown flies. (D) The knockdown of *GmmSRPN10* results in a small but significant decrease in PF midgut infection, suggesting that the expression of GmmSRPN10 is important for PF survival (each data point represents a biological replicate, with a total of >300 flies dissected scored for infection per treatment).

For GmmSRPN10 to inactivate bloodmeal complement cascade serine proteases, it should be secreted into the midgut lumen. Peptide sequence analysis using SignalP 3.0 [42] predicted that GmmSRPN10 lacked an N-terminal signal peptide associated with the secretory pathway, while SecretomeP 2.0 [43] prediction suggests that GmmSRPN10 has sequence elements indicative of non-classically secreted proteins (S4 Fig.). As such, we questioned if GmmSRPN10 could be secreted via the non-classical pathway without a leader sequence [44], [45]. To determine if GmmSRPN10 localises to the midgut lumen, rabbit α-GmmSRPN10 antisera was raised by immunising rabbits with a short, immunogenic peptide specific to GmmSRPN10 (S5A Fig.). The antisera recognised and bound to His:GmmSRPN10 in a concentration dependent manner (S5B Fig.). Probing of harvested tsetse midguts with IgG-enriched antiserum identified GmmSRPN10 (migrating at approximately 40 kDa) both in midgut tissues and contents washed from the midgut lumen (Fig. 5A, S6 Fig.). This suggests that GmmSRPN10, as it is secreted into the midgut lumen, comes into contact with an ingested bloodmeal.

We next attempted to verify that the trypanosome infection phenotype observed in flies fed with ds*GmmSRPN10* was indeed caused by depletion of both the transcript and the protein. Experimental tsetse fed with dsRNA were infected with trypanosomes and subsequently dissected at day 10 post-infection. In conjunction with scoring for the percentage of infected midguts, tissue samples were collected and subjected to qPCR and Western analyses. Using *Glossina α-tubulin* as the reference gene, we determined that *GmmSRPN10* transcript abundance was decreased in ds*GmmSRPN10*-fed midguts compared to controls fed with ds*EGFP* (Fig. 5B). This decrease in *GmmSRPN10* transcript level was significant compared to that of *β-tubulin*, which remained relatively unchanged (fold change ∼1) regardless of dsRNA treatment. Western blotting using α-GmmSRPN10 also confirmed a decrease in protein expression in the same pool of knockdown midguts used for qPCR (Fig. 5C).

The infection rate of trypanosomes in *GmmSRPN10*-knockdown tsetse fell by one third compared to ds*EGFP*-fed controls (Fig. 5D). This reduced rate of midgut infections was statistically significant (non-parametric t-test, P = 0.0137) across 8 biological replicates with a total of more than 300 flies dissected for each treatment. As the experimental tsetse are fed with fresh blood once every two days post-infection, this demonstrates a correlation between reduced PF infection in the presence of bloodmeal complement upon *GmmSRPN10* knockdown. Together with the *in vitro* assays involving complement cascade serine protease inhibition, these data suggest that one function of GmmSRPN10 is to act as a complement inhibitor in the tsetse midgut, which PF trypanosomes use to evade lysis by the serum complement introduced into the midgut environment with every bloodmeal.

## Discussion

Blood-borne pathogens typically evolve an intimate life-cycle with haematophagous arthropods that inadvertently become their vectors for transmission. As such, pathogens can often develop extensive interactions with their arthropod vectors where they modify their feeding behaviour or exploit pre-existing biological secretions from the vector to improve their chances of survival and transmission [8], [36], [46], [47]. Here we described how PF trypanosomes benefit from the activity of tsetse serpin, GmmSRPN10, to avoid lysis by bloodmeal complement when in the fly's midgut.

Phylogenetic analysis suggests that GmmSRPN10, along with three other tsetse serpins, may not have evolved in a common blood feeding ancestor, but may have evolved independently after speciation from the last common ancestor. While all four of the investigated serpins showed causality between gene knockdown and decreased infection of trypanosomes in tsetse, only GmmSRPN10 could be correlated to the inactivation of complement cascade serine proteases by activity assays utilising a recombinant version of the protein. Inactivation of both human rC1s and rFactor D, initiating serine proteases of the classical and alternative pathways, respectively [48], suggests that GmmSRPN10 may have a generic inhibitory function against complement cascade serine proteases. This might be a characteristic of arthropod complement-inhibitory factors, as exhaustive investigation into scabies mite serine proteases (SMB3, SMB4) as well as inactive serine proteases have found them to inhibit all three complement cascade pathways [8], [49].

For GmmSRPN10 to function as an inhibitor of bloodmeal complement, it should be secreted into the midgut lumen where it can come into direct contact with the resting bloodmeal. Unlike other documented complement-inhibiting arthropod serpins [8], [49], GmmSRPN10 does not have an N-terminal signal peptide to identify it as a protein secreted via the classical pathway. However, the lack of an N-terminal signal sequence is not definitive evidence that a protein is not secreted, as analyses of other biological systems indicate that proteins without an N-terminal signal peptide can be present at comparable, if not higher, levels in the secretome compared to proteins with an N-terminal signal peptide [50], [51]. Our Western blots detected GmmSRPN10 in the tsetse midgut lumen in both teneral and non-teneral flies. As teneral flies are newly emerged and have never fed on blood, this suggests that GmmSRPN10 is constitutively secreted into the midgut lumen of emergent tsetse in preparation for feeding.

A recent analysis of transcriptome fold changes within tsetse SGs identified GmmSRPN10 (annotated as Serpin 6, GeneBank ID ABC25076) as amongst an exclusive subset of genes up-regulated during chronic trypanosome infection [52]. Although implicated as a possible modulator of tsetse immunity, our knockdown experiments suggest that it is unlikely to fit its suggested role as a positive regulator of fly immunity in the midgut environment. A possible reason for up-regulation of GmmSRPN10 in trypanosome-infected SGs may be related to the changes in feeding behaviour exhibited by these flies. Tsetse with infected SG take longer to probe and feed [53], and this may necessitate increased expression of GmmSRPN10. Regardless, expression in the tsetse SG suggests inactivation of complement in the bloodmeal may already begin prior to ingestion and continue in the resting bloodmeal within the tsetse midgut. Although we are currently uncertain how dsRNA feeding affects GmmSRPN10 expression in the SG, gene knockdown by dsRNA feeding has been shown to be specific to genes found in the midgut [28]. Likewise, we are uncertain regarding the knockdown status of *GmmSRPN10* in the SG. However our data demonstrates that *GmmSRPN10* knockdown on the transcript and protein levels is consistent in the midgut with dsRNA feed, and this leads to a decrease in the rate of infection of *T. brucei* in tsetse.

Collectively, our findings suggest that GmmSRPN10 can inactivate the complement cascade by inhibiting the activity of cascade activators present in the ingested bloodmeal. When establishing PF trypanosomes encounter a fresh bloodmeal, the complement inhibitory activity of GmmSRPN10 appears to confer protection for these trypanosome PFs, as a decrease in GmmSRPN10 lowers the success rate of PF midgut infection. While all tsetse midgut serpins appear to be important for PF midgut survival, direct inhibition of complement cascade serine proteases could only be confirmed with GmmSRPN10. Therefore, we are currently unable to discern if the reduction in midgut PF infection with knockdown of the other three serpins is indeed related to the loss of complement inhibition, or if the impact on PF survival from *GmmSRPN10* knockdown alone is moderated by the redundancy of another three serpins of similar function.

While exploitation of complement-inactivating proteins in vectors by the pathogens has previously been described in other pathogen-vector relationships [9], [15], it is the first time this phenomenon has been reported in African trypanosomes and tsetse flies. Our experiments confirm the sensitivity of PFs to complement lysis by fresh serum and they likewise reveal that midgut-infecting PFs benefit in a small but significant manner from a complement-deactivating serpin secreted by the fly. It remains unresolved why the tsetse would secrete a serpin that favours a parasitic infection, but investigations into complement-inhibiting molecules secreted by other haematophagous arthropods suggest that this may be part of a strategy to minimise damage to the midgut epithelium or peritrophic matrix by bloodmeal complement during feeding [5], [6].

As other trypanosomatids, such as *Trypanosoma cruzi*, have been shown to express innate complement-inactivating factors [54], we cannot discount that similar factors exist within *T. brucei*. One possible complement-evasion mechanism that may be innate to PF *T. brucei* and *T. congolense* involves sequestering sialic acids (SA) from red blood cells, which could consequently mask trypanosomes from activating the complement cascade. *T. brucei* PFs defective in the expression glycosylphosphatidylinositol (GPI)-anchored trans-sialidases, do not survive in the tsetse midgut and this may be due to the inability of these mutant parasites to transfer sialic acids to their cell surface GPI acceptor molecules [55]. However, there is currently no direct evidence suggesting that the transfer of SA or the expression of TS itself is related to a complement evasion strategy.

Furthermore, *T. brucei* crosses the tsetse peritrophic matrix (PM) on at least two occasions during the course of infection in the fly. This occurs once in the posterior midgut to gain access to the ectoperitrophic space located between the PM and the midgut epithelium, and a second time in the anterior midgut upon trypanosome re-entry into the lumen of the alimentary canal [18], [56]–[58]. Why trypanosomes would exhibit this presumably energetically costly behaviour is unknown, but our findings suggest this may be related to the attempts by complement-susceptible PFs to escape fresh bloodmeal content in the midgut lumen. As secreted proteins can be found at higher concentrations in proximity to epithelial tissue (as in the case of mammalian intestinal anti-microbial peptides [59]), we postulate that PM crossing may also be due to PFs seeking regions of high tsetse serpin concentration as further protection against complement lysis.

In conclusion, evasion of bloodmeal complement by *T. brucei* PFs by taking advantage of tsetse biology illustrates the complicated relationship African trypanosomes share with the tsetse, as well as the innovative evolutionary adaptations in blood borne parasites to achieve cyclical transmission within their insect vectors.

## Supporting Information

S1 Fig **There is no clear evolutionary relationship in the RCL of serpins in haematophageous insects.** Phylogenetic trees for representative insect serpins generated in parellel using (A) full length sequence or (B) RCL sequence did not resolve a clear evolutionary relationship of serpins in insects adapted to blood feeding. Bootstrap values are presented and tsetse serpins are denoted with *.
(TIF)

S2 Fig **Representative gel of RT-PCR results of tsetse serpin transcript levels with dsRNA feeding.** Amplicons generated using RT-PCR from total mRNA extracted from the midgut tissue of experimental tsetse fed with dsRNA with different gene targets (*GmmSRPN3*, *GmmSRPN10*, *GmmSRPN5*, *GmmSRPN9* and *EGFP*). The relative intensity of bands generated from primers targeting *GmmSRPN*s were normalised against the band intensity for *GAPDH* for each dsRNA treatment. The normalised band intensity for each transcript was subsequently presented as a % (S1 Table) of the normalised band intensity of the corresponding band generated from mRNA extracted from *EGFP* dsRNA-fed tsetse.
(TIF)

S3 Fig **His::GmmSRPN10 is an inhibitory serpin.** (A) Mass spectrometry analysis of ∼40 kDa and ∼37 kDa fractions of recombinant His::GmmSRPN10 confirms the ∼40 kDa fraction is the full length protein, while the ∼37 kDa protein represents a C-terminal truncation of His::Serpin10 at the reactive centre loop. (B) Recombinant His::GmmSRPN10 can inhibit trypsin activity in a concentration dependent manner.
(TIF)

S4 Fig **SignalP and SecretomeP analysis of GmmSRPN10.** (A) SignalP analysis did not detect a signal peptide (*S-score*) nor a signal peptide cleavage site (*C-score*) either independently or as a geometric average (*Y-score*). (B) SecretomeP analysis GmmSRPN10 indicates an NN-score above the threshold of 0.5. In the absence of a signal peptide, this suggests that GmmSRPN10 is secreted in a non-classical manner.
(TIF)

S5 Fig **Generation of antibody against GmmSRPN10.** (A) The immunising peptide used to to generate a polyclonal α-GmmSRPN10 rabbit antiserum, represents positions 184–199 of the GmmSRPN10 protein sequence. The predicted region with highest immunogenicity is shaded in grey. (B) Western blotting against recombinant His::GmmSRPN10 at decreasing protein concentrations (1 = 2.4 µg protein, 2 = 0.8 µg protein, 3 = 0.6 µg protein) demonstrates that Protein G-purified α-GmmSRPN10 antiserum is specific for GmmSRPN10.
(TIF)

S6 Fig **Western blots showing GmmSRPN10 is detected in tsetse midgut tissue and washed lumen content.** Midgut (MG) tissue was collected from teneral (unfed) flies and flies receiving one bloodmeal. Midgut lumen content was washed out with PBS and both collected tissue and lumen content were resolved on 12.5% SDS-PAGE gel prior to blotting with α-GmmSRPN10 rabbit antiserum. (A) Midgut tissue lysate (2, 3, 4, 5 MG equivalents) and (B) midgut lumen content (1 and 2 MG equivalents) were isolated from teneral and fed flies. GmmSRPN10 is present in the MG lumen at comparable levels to MG tissue. PVDF membrane staining with nigrosine is shown to indicate protein loading.
(TIF)

S1 Table **Relative band intensity (%) for** ***GmmSRPN3*** **,** ***GmmSRPN5*** **,** ***GmmSRPN9*** **and** ***GmmSRPN10*** **in from RT-PCR amplicon products on midgut RNA extracted from dsRNA treated flies.**
(XLSX)

S2 Table **BLAST alignment of tsetse serpin dsRNA fragments.** Output from multi-alignment BLAST of dsRNA fragments targeting tsetse serpins against all serpin gene sequences. Each dsRNA fragment only has significant alignment against its target gene.
(DOCX)

S3 Table **Rare codons coding for putative tsetse midgut serpins.** All putative tsetse midgut serpins were coded with eukaryotic-specific codons. Only expression of *GmmSRPN10*, the gene with the lowest percentage of rare codons, was possible using a bacterial expression system.
(DOCX)
