# Supplementary figures and images for: Tsetse GmmSRPN10 Has Anti-complement Activity and Is Important for Successful Establishment of Trypanosome Infections in the Fly Midgut
Source: PLoS Negl Trop Dis. 2015 Jan 8;9(1):e3448. doi: 10.1371/journal.pntd.0003448 (PMC4287558; doi:10.1371/journal.pntd.0003448)

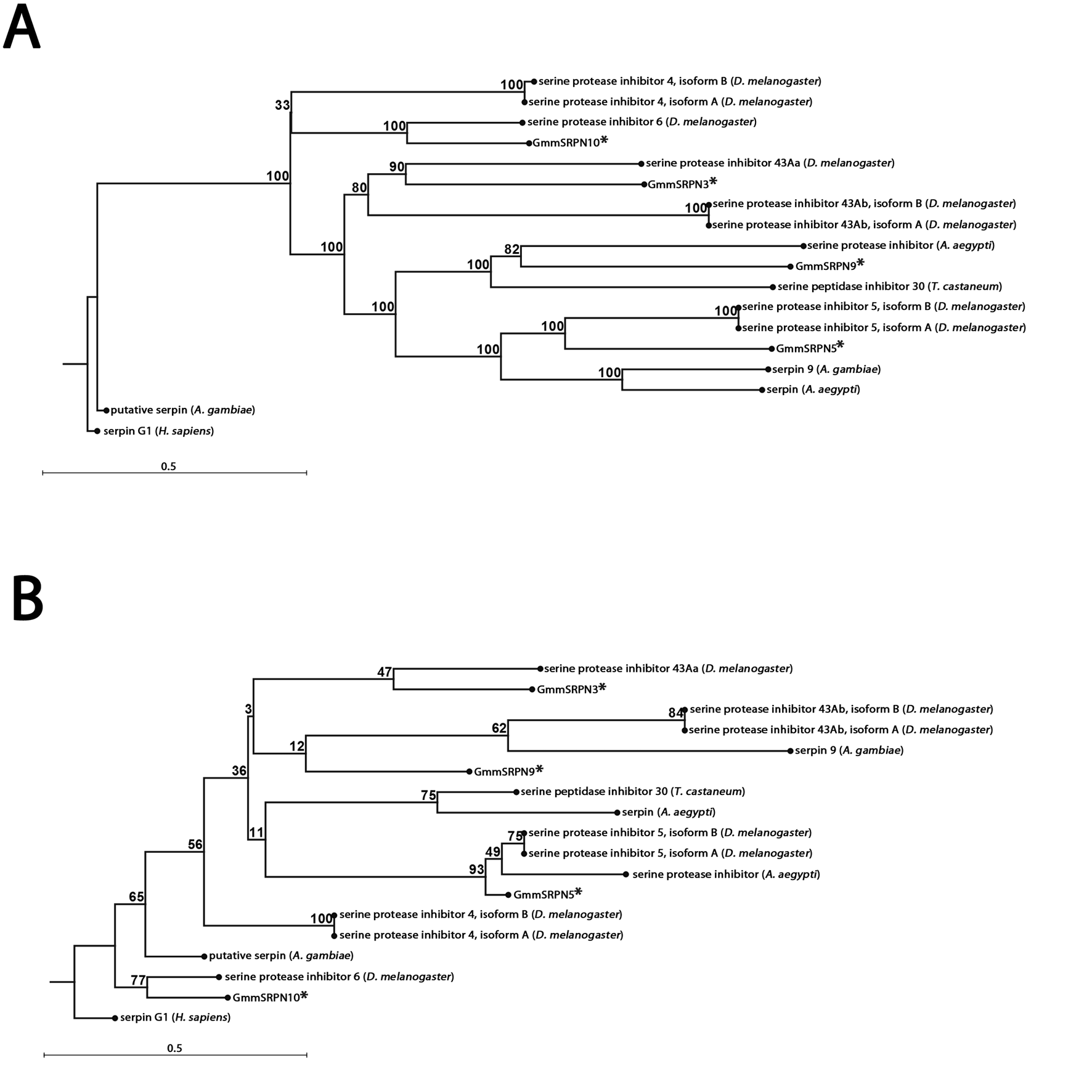

Supplement: S1 Fig — There is no clear evolutionary relationship in the RCL of serpins in haematophageous insects. Phylogenetic trees for representative insect serpins generated in parellel using (A) full length sequence or (B) RCL sequence did not resolve a clear evolutionary relationship of serpins in insects adapted to blood feeding. Bootstrap values are presented and tsetse serpins are denoted with *. (TIF) [file pntd.0003448.s001.tif]

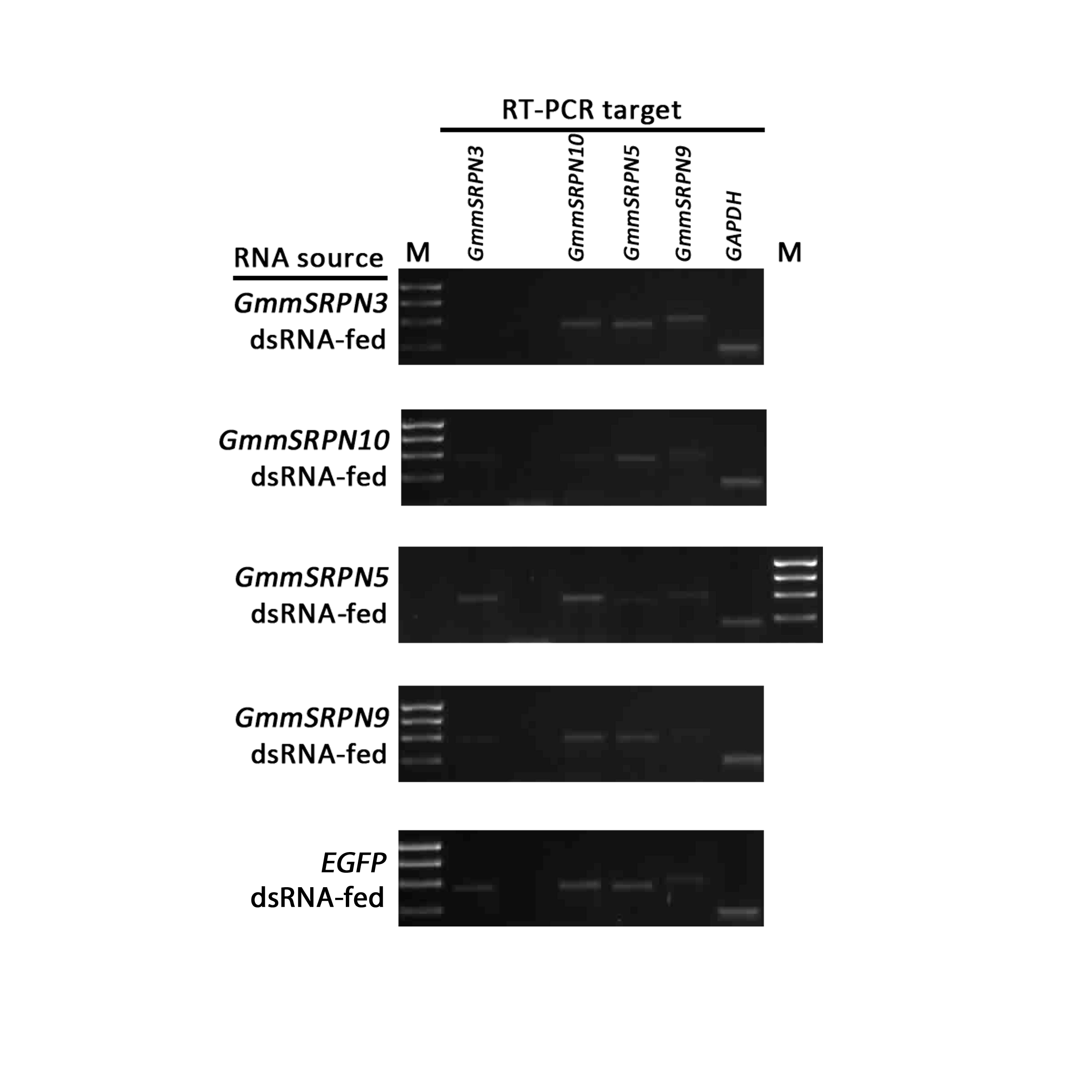

Supplement: S2 Fig — Representative gel of RT-PCR results of tsetse serpin transcript levels with dsRNA feeding. Amplicons generated using RT-PCR from total mRNA extracted from the midgut tissue of experimental tsetse fed with dsRNA with different gene targets (GmmSRPN3, GmmSRPN10, GmmSRPN5, GmmSRPN9 and EGFP). The relative intensity of bands generated from primers targeting GmmSRPNs were normalised against the band intensity for GAPDH for each dsRNA treatment. The normalised band intensity for each transcript was subsequently presented as a % (S1 Table) of the normalised band intensity of the corresponding band generated from mRNA extracted from EGFP dsRNA-fed tsetse. (TIF) [file pntd.0003448.s002.tif]

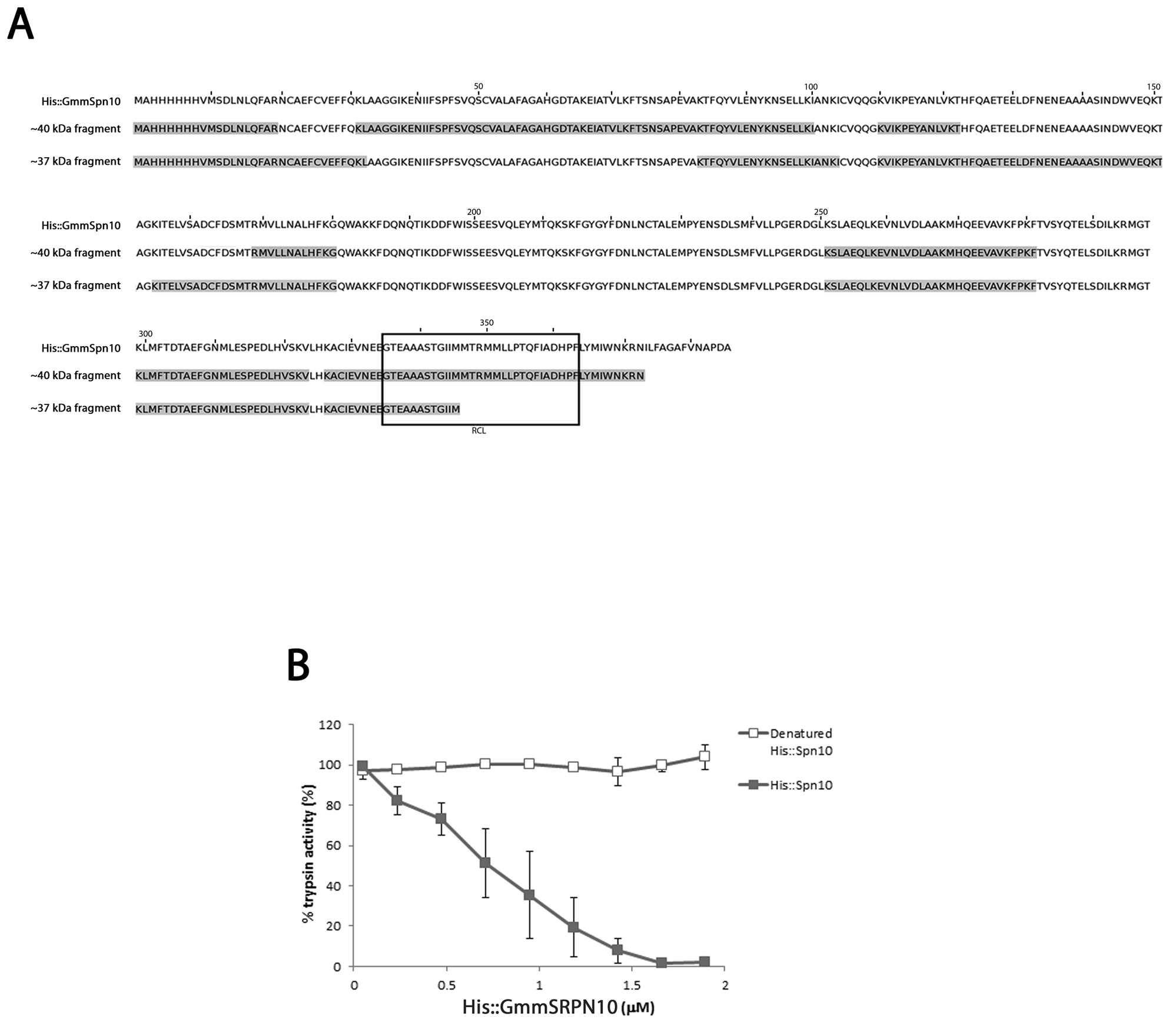

Supplement: S3 Fig — His::GmmSRPN10 is an inhibitory serpin. (A) Mass spectrometry analysis of ∼40 kDa and ∼37 kDa fractions of recombinant His::GmmSRPN10 confirms the ∼40 kDa fraction is the full length protein, while the ∼37 kDa protein represents a C-terminal truncation of His::Serpin10 at the reactive centre loop. (B) Recombinant His::GmmSRPN10 can inhibit trypsin activity in a concentration dependent manner. (TIF) [file pntd.0003448.s003.tif]

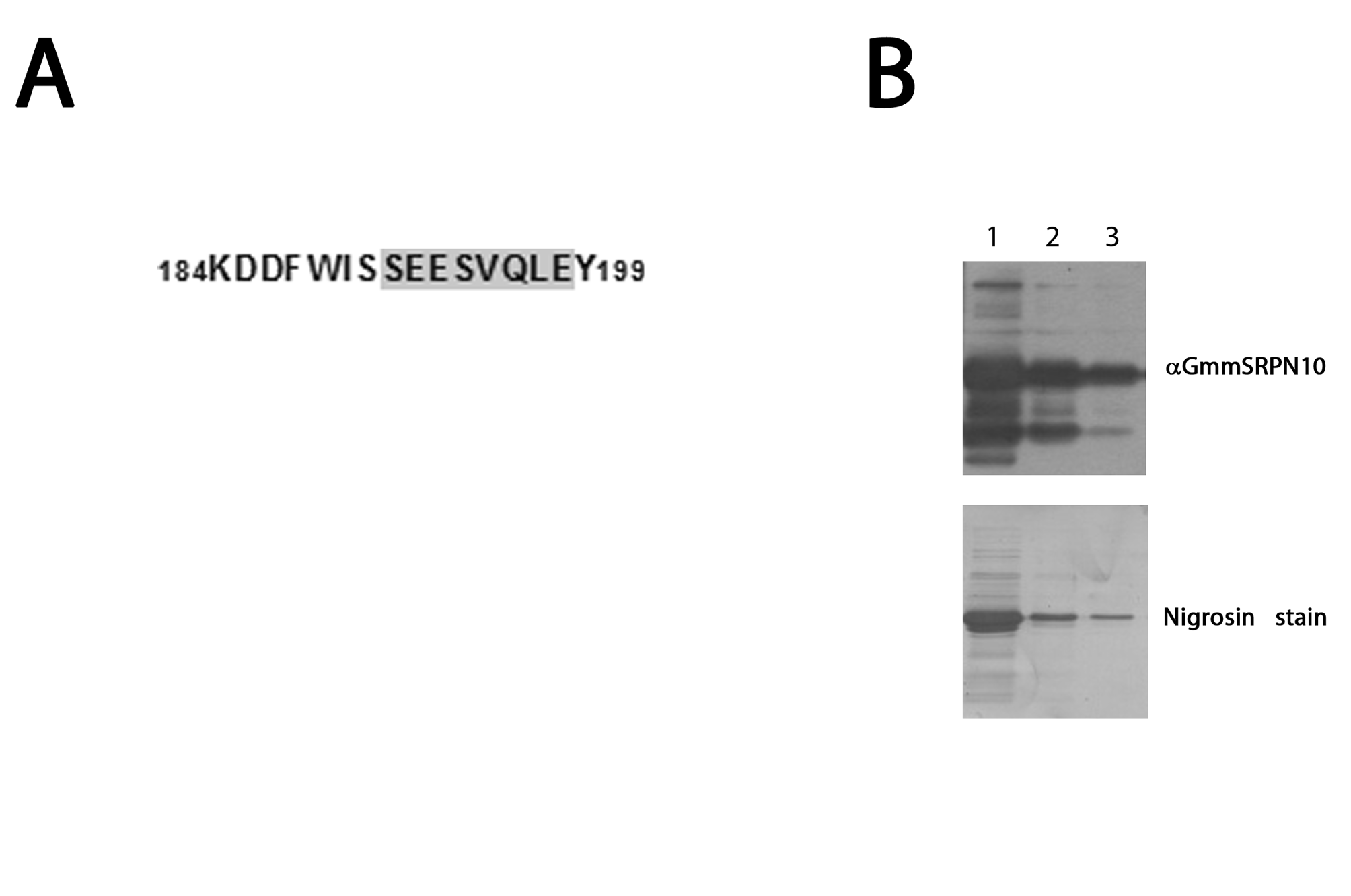

Supplement: S5 Fig — Generation of antibody against GmmSRPN10. (A) The immunising peptide used to to generate a polyclonal α-GmmSRPN10 rabbit antiserum, represents positions 184–199 of the GmmSRPN10 protein sequence. The predicted region with highest immunogenicity is shaded in grey. (B) Western blotting against recombinant His::GmmSRPN10 at decreasing protein concentrations (1 = 2.4 µg protein, 2 = 0.8 µg protein, 3 = 0.6 µg protein) demonstrates that Protein G-purified α-GmmSRPN10 antiserum is specific for GmmSRPN10. (TIF) [file pntd.0003448.s005.tif]

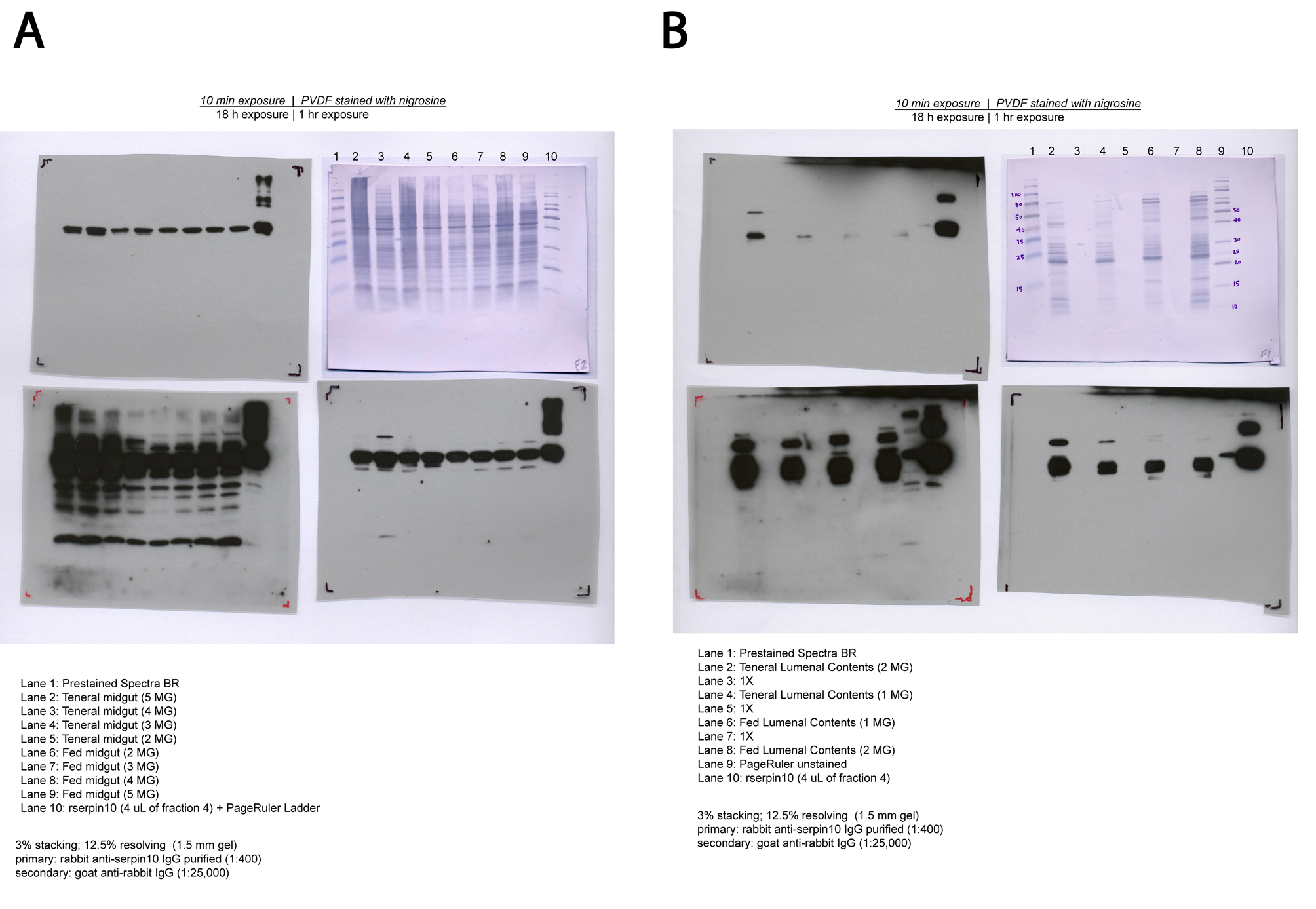

Supplement: S6 Fig — Western blots showing GmmSRPN10 is detected in tsetse midgut tissue and washed lumen content. Midgut (MG) tissue was collected from teneral (unfed) flies and flies receiving one bloodmeal. Midgut lumen content was washed out with PBS and both collected tissue and lumen content were resolved on 12.5% SDS-PAGE gel prior to blotting with α-GmmSRPN10 rabbit antiserum. (A) Midgut tissue lysate (2, 3, 4, 5 MG equivalents) and (B) midgut lumen content (1 and 2 MG equivalents) were isolated from teneral and fed flies. GmmSRPN10 is present in the MG lumen at comparable levels to MG tissue. PVDF membrane staining with nigrosine is shown to indicate protein loading. (TIF) [file pntd.0003448.s006.tif]
