# Supplementary material for: Tsetse GmmSRPN10 Has Anti-complement Activity and Is Important for Successful Establishment of Trypanosome Infections in the Fly Midgut
Source: PLoS Negl Trop Dis. 2015 Jan 8;9(1):e3448. doi: 10.1371/journal.pntd.0003448 (PMC4287558; doi:10.1371/journal.pntd.0003448)
Supplement: S2 Table — BLAST alignment of tsetse serpin dsRNA fragments. Output from multi-alignment BLAST of dsRNA fragments targeting tsetse serpins against all serpin gene sequences. Each dsRNA fragment only has significant alignment against its target gene. (DOCX) [file pntd.0003448.s008.docx]

| **ID** | **Significant alignment** | **Max score** | **Total score** | **% Query cover** | **E value** | **% Identity** | **Accession** |
| --- | --- | --- | --- | --- | --- | --- | --- |
| GmSRPN3_dsRNA | GmmSRPN3 | 996 | 996 | 100 | 0.0 | 100 | 30184 |
| GmSRPN5_dsRNA | GmmSRPN5 | 983 | 983 | 100 | 0.0 | 100 | 43035 |
| GmmSRPN9_dsRNA | GmmSRPN9 | 1077 | 1077 | 100 | 0.0 | 100 | 58848 |
| GmmSRPN10_dsRNA | GmmSRPN10 | 990 | 990 | 100 | 0.0 | 100 | 1853 |
