# Supplementary material for: Tsetse GmmSRPN10 Has Anti-complement Activity and Is Important for Successful Establishment of Trypanosome Infections in the Fly Midgut
Source: PLoS Negl Trop Dis. 2015 Jan 8;9(1):e3448. doi: 10.1371/journal.pntd.0003448 (PMC4287558; doi:10.1371/journal.pntd.0003448)
Supplement: S3 Table — Rare codons coding for putative tsetse midgut serpins. All putative tsetse midgut serpins were coded with eukaryotic-specific codons. Only expression of GmmSRPN10, the gene with the lowest percentage of rare codons, was possible using a bacterial expression system. (DOCX) [file pntd.0003448.s009.docx]

| **Gene** | **Amino acid** | **Rare Codon** | **Number** | **% total sequence** |
| --- | --- | --- | --- | --- |
| *GmmSRPN3* | Arginine (R) | AGA | 2 | 6.2 |
|  |  | AGG | 1 |  |
|  |  | CGA | 2 |  |
|  |  | CGG | 2 |  |
|  | Glycine (G) | GGA | 3 |  |
|  | Isoleucine (Ile) | AUA | 8 |  |
|  | Leucine (L) | CUA | 4 |  |
|  | Proline (P) | CCC | 3 |  |
| *GmmSRPN5* | Arginine (R) | AGA | 2 | 6.7 |
|  |  | AGG | 0 |  |
|  |  | CGA | 5 |  |
|  |  | CGG | 2 |  |
|  | Glycine (G) | GGA | 2 |  |
|  | Isoleucine (Ile) | AUA | 12 |  |
|  | Leucine (L) | CUA | 2 |  |
|  | Proline (P) | CCC | 3 |  |
| *GmmSRPN9* | Arginine (R) | AGA | 3 | 7.8 |
|  |  | AGG | 0 |  |
|  |  | CGA | 3 |  |
|  |  | CGG | 1 |  |
|  | Glycine (G) | GGA | 7 |  |
|  | Isoleucine (Ile) | AUA | 9 |  |
|  | Leucine (L) | CUA | 6 |  |
|  | Proline (P) | CCC | 5 |  |
| *GmmSRPN10* | Arginine (R) | AGA | 2 | 5.8 |
|  |  | AGG | 0 |  |
|  |  | CGA | 1 |  |
|  |  | CGG | 0 |  |
|  | Glycine (G) | GGA | 3 |  |
|  | Isoleucine (Ile) | AUA | 6 |  |
|  | Leucine (L) | CUA | 6 |  |
|  | Proline (P) | CCC | 4 |  |
